# Enhancing Bioavailability and Stability of Plant Secondary Metabolites: Formulation and Characterization of Nanophytosomes Encapsulating Red Bryony and Horned Poppy Extracts

**DOI:** 10.3390/jfb16060194

**Published:** 2025-05-24

**Authors:** Atoosa Olfati, Naser Karimi, Elham Arkan, Mohsen Zhaleh, M. R. Mozafari

**Affiliations:** 1 Department of Nano-Biotechnology, Faculty of Innovative Science and Technology, Razi University, Kermanshah 6714967346, Iran; a.olfati71@gmail.com; 2 Department of Biology, Faculty of Science, Razi University, Kermanshah 6715847141, Iran; 3 Nano Drug Delivery Research Center, Health Technology Institute, Kermanshah University of Medical Sciences, Kermanshah 6715847141, Iran; elhamarkan@yahoo.com; 4 Department of Anatomical Sciences, School of Medicine, Kermanshah University of Medical Sciences, Kermanshah 6715847141, Iran; zhalehmohsen@yahoo.com; 5 Australasian Nanoscience and Nanotechnology Initiative (ANNI), Monash University LPO, Clayton, VIC 3800, Australia

**Keywords:** bioavailability, herbal extract, Horned Poppy, nanophytosome, Red Bryony, drug delivery

## Abstract

Biocompatible nanocarriers were formulated by encapsulating medicinal extracts from *Bryonia dioica* (Red Bryony) and *Glaucium leiocarpum* (Horned Poppy) using a nanophytosome approach. The nanophytosomes were prepared by employing a thin-film hydration technique. The SEM results showed a broad size distribution for both nanophytosomes, and the encapsulation efficiency was about 75–80% for both Red Bryony and Horned Poppy nanophytosomes, as confirmed through scanning electron microscopy (SEM) and dynamic light scattering (DLS). Zeta potential analysis indicated sufficient surface charges to maintain colloidal stability. Encapsulation improved the release characteristics of the extracts, exhibiting an initial burst release followed by sustained release, which is advantageous for enhancing bioavailability within a liquid environment. Fourier-transform infrared (FTIR) spectroscopy identified key functional groups, confirming the successful encapsulation of bioactive ingredients within the nanophytosomes. Cytotoxicity tests on fibroblast cell lines (HSF-PI 16) demonstrated the safety of these nanocarriers, indicating biocompatibility at concentrations up to 200 μg/mL. Stability tests over 30 days revealed minimal size fluctuations, further supporting the structural integrity of the formulations. Results suggest that the synthesized nanophytosomes could serve as effective and novel nanocarriers for herbal delivery, addressing the bioavailability limitations of herbal extracts and offering a promising approach for therapeutic applications in both traditional and alternative medicine. This is the first study to report nanophytosome-based delivery of Red Bryony and Horned Poppy extracts.

## 1. Introduction

Medicinal plants hold significant value in traditional medicine across the globe. Numerous reliable sources have examined and researched the medicinal properties of plants in relation to various diseases [1,2]. These plants have attracted attention due to their low side effects, high availability of herbal sources, affordability, and numerous therapeutic properties [3]. However, it is important to recognize that the healing potential of many plants remains unexplored or inadequately studied. Notable examples of such plants include Red Bryony (*Bryonia dioica Jacq.* RB) and Horned Poppy (*Glaucium leiocarpum Boiss*. HP).

RB, a member of the Cucurbitaceae family characterized by its durable nature and tuberous root system, possesses a substantial history of therapeutic application extending back to ancient and medieval eras [4]. Research indicates that the therapeutic qualities of this plant are linked to its tuberous root, which is utilized for a variety of conditions, including chronic rheumatism, epilepsy, and as a laxative and emetic. Recent studies, however, have demonstrated the antioxidant, anti-diabetic, anti-inflammatory, analgesic, and anti-cancer properties of both the root and fruit of this valuable plant. Additionally, some sources have noted the potential toxic effects associated with it [4,5]. HP belongs to the genus Glaucium and the Papaveraceae family. This plant genus contains various alkaloids that offer numerous medicinal benefits, including anticancer and anticholinesterase properties. Research indicates that aporphine alkaloids are most prevalent in these plants. However, it appears that many species within this genus have not been extensively researched for their phytochemical and medicinal properties [6].

Medicinal properties in plants arise from secondary metabolites, such as phenolic compounds, terpenoids, and nitrogenous cyclic compounds, which are primarily polar and soluble molecules [7,8]. These compounds often struggle to passively cross cell membranes due to their size or low-fat solubility, resulting in low bioavailability and susceptibility to degradation under adverse conditions (e.g., oxygen, temperature, pH) [8]. To enhance the absorption and bioavailability of these effective plant compounds, various carriers, particularly nanocarriers, are employed [9]. Techniques like emulsification and nanophytosomes facilitate the passage of these compounds through biological membranes and blood barriers. Additionally, their nano dimensions reduce the likelihood of recognition and elimination by the body’s systems, such as the kidneys and the liver, thereby prolonging the drug’s effects and reducing required dosages [10,11].

Various nanocarriers, including liposomes, niosomes, solid lipid nanophytosomes (SLNs), and carbon nanotubes, have been utilized in drug delivery [12]. Among these, nanophytosomes are gaining attention due to their potential for commercialization and low-cost mass production, as well as their liposome-like structure [12]. Nanophytosomes are formed by combining plant extracts or phytochemicals with phospholipids, addressing challenges like solubility limits, cell membrane permeability, adverse effects of plant extracts, and enhancing bioavailability and targeted delivery [13]. Research indicates that nanophytosomes are more stable than other structures and feature a unique loading mechanism, where the drug forms strong hydrogen bonds with the hydrophilic choline head in lecithin, improving drug trapping efficiency. Additionally, the phosphatidylcholine in nanophytosomes is highly soluble in bile, contributing to liver tissue repair [14,15,16].

Previous research has demonstrated the dissolution and intestinal permeability of diosmin. Further investigation indicated that nanophytosomes, exhibiting particle sizes of approximately 316 nm, significantly enhanced drug transport capability to 99%. These nanophytosomes also improved both the physicochemical stability and dissolution characteristics of the diosmin [17]. In another study, nanophytosome formulations were utilized to enhance the anti-diabetic properties of chrysin, a flavonoid, and facilitate glucose absorption in muscle cells. This formulation, measuring 117 nm, achieved a trapping efficiency of 99%, suggesting enhanced glucose uptake in muscle cells compared to chrysin alone [18]. A recent study explored the potential of nanophytosomes to improve the oral absorption and solubility of silymarin. The research successfully synthesized vesicles with an average size of 218 nm. This nanoencapsulation technique resulted in a significant enhancement of drug content, reaching up to a 90% improvement compared to the unencapsulated form, suggesting a promising avenue for enhanced silymarin bioavailability [19]. However, the impact of nanophytosomes on enhancing the bioavailability and transfer efficiency of various plant compounds, such as RB and HP, has yet to be explored. Therefore, it is crucial to investigate the potential of these novel nanocarriers. The objective of this research is to develop a safe and stable nanocarrier that boosts bioavailability, facilitates efficient transfer, and enhances the productivity of RB and HP extracts. To achieve this, nanophytosomes were formulated, and their physicochemical properties, including particle size, zeta potential, drug loading, encapsulation efficiency, morphology, release profile, stability, and cytotoxicity on fibroblast cells, were thoroughly examined. The findings from this study contribute to the design of innovative nanocarriers with multiple advantages, offering an effective approach to improving the bioavailability and stability of plant-derived compounds.

## 2. Materials and Methods

### 2.1. Chemicals and Reagents

Soy lecithin with a purity exceeding 99%, chloroform, ethanol, and dimethyl sulfoxide (DMSO), along with Whatman filter paper (No. 1), were sourced from Merck (Darmstadt, Germany). Additionally, dialysis bags, 96-well plates, phosphate-buffered saline (PBS), DMEM (Dulbecco’s Modified Eagle Medium) culture medium, amphotericin, streptomycin, and penicillin were obtained from Bioideh (Tehran, Iran). The MTT reagent used in the cytotoxicity evaluations was acquired from Sigma (Sigmaringen, Germany). Fibroblast cell lines were obtained from the Pasteur Institute of Iran (IPI). The medicinal plants RB and HP were collected from Kermanshah province, Iran, and their botanical identification was conducted by the biology department at Razi University of Kermanshah, Iran, where their exactness was confirmed.

### 2.2. Preparation of the RB and HP Extracts

To prepare the medicinal plant extracts, 100 g of RB root and shoots and 100 g of HP shoots were dried and ground. A mixture of absolute ethanol and distilled water was added in a 70:30 (*v*/*v*) ratio and incubated for 18 h. The supernatant was filtered through Whatman No. 1 filter paper and subjected to rotary evaporation to remove the ethanol. The extract was then incubated for 24 h to obtain a dry powder, which was stored in the dark at −20 °C for future use [20].

### 2.3. Preparation of the Nanophytosomes

The process of the thin-film hydration method was used to prepare the nanophytosomes. Soy lecithin and dry powdered plant extracts were combined in a 2:1 ratio in chloroform and incubated at 4 °C for 24 h to create this nanocarrier. It should be mentioned that an appropriate solvent was added to the herbal plant extract if it was initially insoluble in chloroform. After adding 2 mL of ethanol solvent to the HP extract, it was added to the solution containing lecithin and chloroform. The produced solution was put into the round-bottom flask of the rotating apparatus at 50 °C and 150 rpm after a 24 h period. Subsequently, a thin layer developed on the round flask’s bottom as the solvent evaporated under the vacuum pump. In the next stage, 50 mL of sterile double-distilled water at 50 °C was added to hydrate the thin layer [21]. At this point, nanophytosomes were formed, but they were still of macro size. To reduce their size to the nanoscale, an Ultrasonic Homogenizer and an Ultrasonic bath were employed according to a procedure described before [22]. The RB and HP nanophytosomes were also treated similarly with the Ultrasonic Homogenizer (UW2070, Bandelin Electronic GmbH & Co. KG, Berlin, Germany) for 2 min, repeated three times with 5 min intervals. This was followed by 20 min treatment in an Elma Ultrasonic bath (D-78224, Elma Schmidbauer GmbH, Berlin, Germany) for RB nanophytosomes and 15 min for HP nanophytosomes.

### 2.4. Evaluation of Drug Loading (DL) and Encapsulation Efficiency (EE)

For the evaluation of drug loading (DL) and encapsulation efficiency (EE) of the nanophytosomes, the loading of herbal extracts into nanophytosomes was assessed using an ultracentrifuge (Beckman Coulter, Optima L-90K) following the method described by Andishmand et al. (2024) [23]. The concentrations of RB and HP in the nanophytosomes were determined using calibration curves, represented by the following equations [23]:

- RB Concentration: y = 0.0081x + 0.0296 (R^2^ = 0.9904)
- HP Concentration: y = 0.0057x + 0.0777 (R^2^ = 0.9957)

After preparing the nanophytosomes loaded with RB and HP extracts, they were centrifuged at 40,000 rpm for 20 min. A 1 mL sample of the supernatant was analyzed via spectrophotometry, and the results were compared to the respective standard curves to calculate drug loading (DL) and encapsulation efficiency (EE) using the following equations [23]:

Drug Loading (DL): DL (%) = (Encapsulated drug/Total weight of nanophytosome) × 100

Encapsulation Efficiency (EE): EE = (amount of loaded drug)/(amount of initial drug) × 100

### 2.5. Evaluation of the Physicochemical Properties of the Nanophytosomes

#### 2.5.1. DLS and Zeta Potential Analysis

To assess the particle size, polydispersity index (PDI), and zeta potential of nanophytosomes, both without and with RB and HP extracts, 1.5 mL of nanophytosome solution was placed in sampling cuvettes for analysis. Particle size was measured using a DLS 2000 (Malvern Instruments Ltd., Herrenberg, Germany), while zeta potential was evaluated with a Zeta Sizer 2000 (Malvern Instruments Ltd., Germany). Measurements were conducted four times at the following conditions: a measurement temperature of 25 °C, a medium viscosity of 0.8872 mPa.s, a medium refractive index of 1.330, and a material refractive index of 0.200.

#### 2.5.2. Scanning Electron Microscopy

To examine the morphology of nanophytosomes loaded with RB and HP extracts, 100 µL of the samples was fixed on a coverslip, dried, and coated with a thin gold layer. The prepared samples were then analyzed using a Fei scanning electron microscope (SEM, Phillips Quanta 450, Springfield, IL, USA).

#### 2.5.3. Fourier Transform Infrared Spectroscopy (FTIR)

FTIR absorption spectra of nanophytosomes with RB and HP extracts, as well as nanophytosomes alone and RB and HP extracts, were analyzed using a Shimadzu FTIR (MIRacle, Japan) over the range of 400 to 4000 cm^−1^. Samples were prepared by mixing the powdered extracts with KBr salt (1:100 ratio), grinding in a glass mortar, and forming a pellet under vacuum for analysis.

### 2.6. Evaluation of the Release Characteristics of the Nanophytosomes

The in vitro release of plant extracts was evaluated using a dialysis bag method in a buffer with pH 7.2 [24]. Following nanophytosomes synthesis and centrifugation, the sediment was resuspended in 1 mL of buffer and placed in a 12 kDa dialysis bag. The release rate was monitored over 24 h, with 1 mL of buffer removed hourly for absorbance measurement at 280 nm (HP) and 268 nm (RB), reported as a percentage with *n* = 3.

### 2.7. Stability Studies

To assess the stability of RB and HP nanophytosomes, samples were stored at room temperature (25 ± 2 °C) for 30 days. Particle size was measured and compared to the baseline values according to a procedure reported earlier [25].

### 2.8. Cytotoxicity Evaluations

Cytotoxicity studies were conducted on the normal human skin fibroblast cell line (HSF-PI 16) according to ISO 10993-5 [26]. The HSF-PI 16 cell line was obtained from the Pasteur Institute of Iran (IPI), provided courtesy of Dr. Faranak Aghaz (School of Pharmacy, Kermanshah University of Medical Sciences). Fibroblast cell lines were cultured in high-glucose Dulbecco’s modified eagle medium (DMEM) supplemented with 10% FBS, amphotericin (0.25 μg/mL), streptomycin (100 μg/mL), and penicillin (100 units/mL), with medium changes every two days to maintain over 80% confluence. Cells were seeded at a density of 2 × 10^3^ cells/well in 96-well plates. The samples tested included nanophytosomes alone, nanophytosomes with RB and HP extracts, and the extracts alone, all at a concentration of 200 μg/L for 24 h. To remove test composite components, media from control and experimental groups were washed with sterile PBS. After adding 50 µL of 5 mg/mL MTT dye to each well and incubating for 4 h at 37°C in a 95% air/5% CO_2_ atmosphere, 200 μL of DMSO was added to dissolve the formazan crystals [26]. Absorbance was measured at 570 nm using a spectrophotometer, and cell viability was calculated as follows [23]:

Cell viability (%) = (A570 (sample))/(A570(control)) × 100

### 2.9. Statistical Analysis

The analysis of data was conducted utilizing Excel 2016 in conjunction with GraphPad Prism software version 10.4.2. The findings are expressed as mean values accompanied by their standard deviations (Mean ± SD). For comparisons among groups, one-way ANOVA was employed, with a significance threshold established at *p* ≤ 0.05. The graphical representations were produced through Prism GraphPad software.

## 3. Results and Discussion

### 3.1. DLS and Zeta Potential of Nanophytosomes

**Figure 1:**
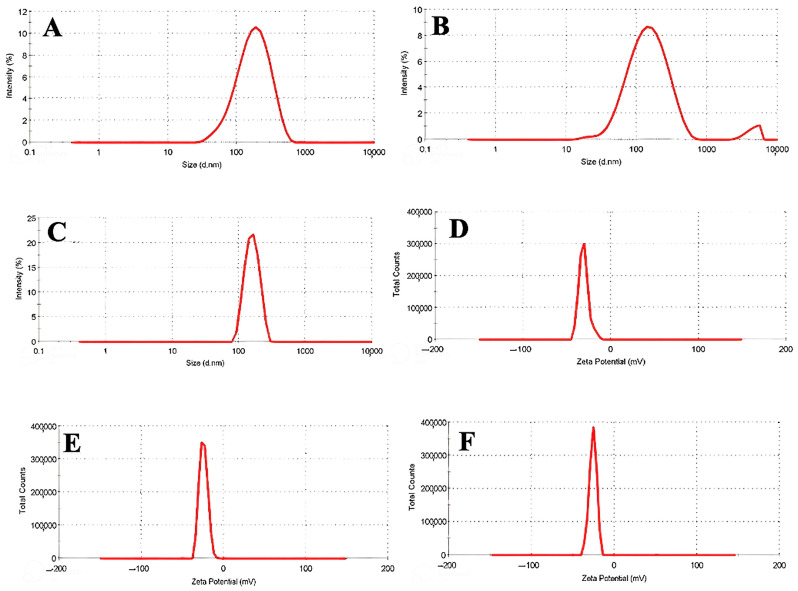
The DLS and zeta potential of nanophytosomes. The DLS of empty nanophytosomes (**A**), the DLS of RB nanophytosomes (**B**), the DLS of HP nanophytosomes (**C**), the zeta potential of empty nanophytosomes (**D**), the zeta potential of RB nanophytosomes (**E**), the zeta potential of HP nanophytosomes (**F**).

In this study, we characterized RB extract-containing nanophytosomes and empty nanophytosomes using DLS and PDI analysis. The DLS results indicated that the RB nanophytosomes had an average size of approximately 148 nm with a PDI of 0.234 (Figure 1B), while the empty nanophytosomes measured 126 nm with a PDI of 0.345 (Figure 1A). The increase in size from 126 to 148 nm upon RB extract addition confirms effective encapsulation, which enhances bioavailability, as particles in the 100–200 nm range are typically well-absorbed by cells [27]. This size increase aligns with prior research indicating that loading bioactive compounds into nanophytosomes generally results in slight size increases.

Regarding PDI, the empty nanophytosomes exhibited a moderately broad distribution with a value of 0.345, while the RB-loaded nanophytosomes showed improved uniformity with a PDI of 0.234. This reduction suggests that the RB extract may stabilize the nanophytosomes, preventing aggregation and leading to more consistent particle sizes. An ideal PDI for loaded nanophytosomes is typically between 0.2 and 0.3, and our findings fit within this range [28]. Previous studies on silymarin-loaded nanophytosomes also reported similar PDI values, indicating that reduced PDI can enhance both bioavailability and long-term stability of well-dispersed nanophytosome systems [29,30].

DLS analysis of the HP extract-containing nanophytosomes revealed a size of 151 nm with a PDI of 0.147 (Figure 1C). This size is notably larger compared to the empty nanophytosomes and RB nanophytosomes, which typically exhibit an increase in size from empty to loaded formulations. The larger size of the HP nanophytosomes can be attributed to the molecular size and structural complexity of the HP extract, confirming effective encapsulation [31]. The literature indicates that bioactive-loaded nanophytosomes generally range from 140 to 200 nm, supporting our findings [28,32]. The HP nanophytosomes exhibited a significantly lower PDI of 0.147 compared to the empty nanophytosomes (0.345) and RB nanophytosomes (0.234), indicating a highly uniform particle size distribution, which is essential for pharmaceutical applications [33]. This narrow distribution suggests that the HP extract may enhance system stability more effectively than the RB extract. A low PDI typically indicates reduced aggregation and improved predictability in biological systems. Previous studies have shown similar low PDI values for various nanophytosome formulations of plant-based polyphenols and flavonoids, highlighting the potential for homogeneous particle distributions [34]. Additionally, research on silymarin-encapsulated nanophytosomes reported PDI values in the range of 0.1–0.2, further supporting the uniformity of our HP nanophytosomes [35].

In this investigation, the zeta potential of RB nanophytosomes was measured as shown in Figure 1E, while the empty nanophytosomes were analyzed according to Figure 1D. The RB nanophytosomes exhibited a less negative zeta potential of −24.5 mV compared to −30 mV for the empty nanophytosomes. Zeta potential is a crucial indicator of colloidal stability, providing insights into particle aggregation tendencies and surface charge. A high zeta potential (above ±30 mV) generally indicates strong electrostatic repulsion, which helps prevent aggregation and enhances stability. The −30-mV zeta potential of the empty nanophytosomes suggests good colloidal stability, indicating sufficient surface charge to repel one another [36,37].

The encapsulation of the RB extract resulted in a slight decrease in zeta potential to −24.5 mV, which, while lower than that of the empty nanophytosomes, still indicates reasonable stability. Similar decreases in zeta potential after loading with plant extracts have been documented in other studies. For instance, nanophytosomes loaded with polyphenolic compounds like resveratrol or curcumin often show reduced zeta potential due to interactions between the bioactive compounds and the lipid bilayer affecting surface chemistry [28,38]. This aligns with findings on quercetin-loaded nanophytosomes, where the zeta potential dropped from −33 mV for empty nanophytosomes to −26 mV for quercetin-loaded ones, consistent with our results [39].

The zeta potential of HP nanophytosomes was measured at −25.7 mV (Figure 1F), which is similar to the −24.5 mV for RB nanophytosomes and −30 mV for empty nanophytosomes. This indicates that HP nanophytosomes have a comparable reduction in surface charge to RB nanophytosomes, although they remain more negative than RB, suggesting different interactions of the HP extract with the nanophytosome surface and potentially better colloidal stability. Although both RB and HP-loaded nanophytosomes exhibited zeta potentials slightly below the ideal threshold of ±30 mV, values around −25 mV are still considered indicative of moderate colloidal stability. This suggests that while the electrostatic repulsion is somewhat reduced due to extract incorporation, the formulations remain sufficiently stable for nanocarrier applications, particularly in systems where additional steric stabilization may occur due to extract components. The zeta potential values for both RB and HP nanophytosomes indicate average colloidal stability, implying that while the extracts slightly reduce electrostatic repulsion, there is still enough surface charge to prevent aggregation under physiological conditions. The observed lower zeta potentials for both HP and RB-loaded nanophytosomes suggest that the extracts partially neutralize the lipid surface charge.

Previous studies have shown similar changes in zeta potential upon encapsulating plant extracts. For example, curcumin-loaded nanophytosomes showed a decrease from −32 mV (empty) to −28 mV (loaded), mirroring the trends seen with RB and HP formulations [40]. Another study on silymarin-loaded nanophytosomes reported a decrease from −34 mV to −27 mV, reinforcing the idea that loading bioactive compounds typically lowers surface charge while maintaining colloidal stability [41]. Overall, both RB and HP formulations demonstrate sufficient stability for drug delivery applications despite the expected decrease in zeta potential after loading.

### 3.2. Scanning Electron Microscopy (SEM)

**Figure 2:**
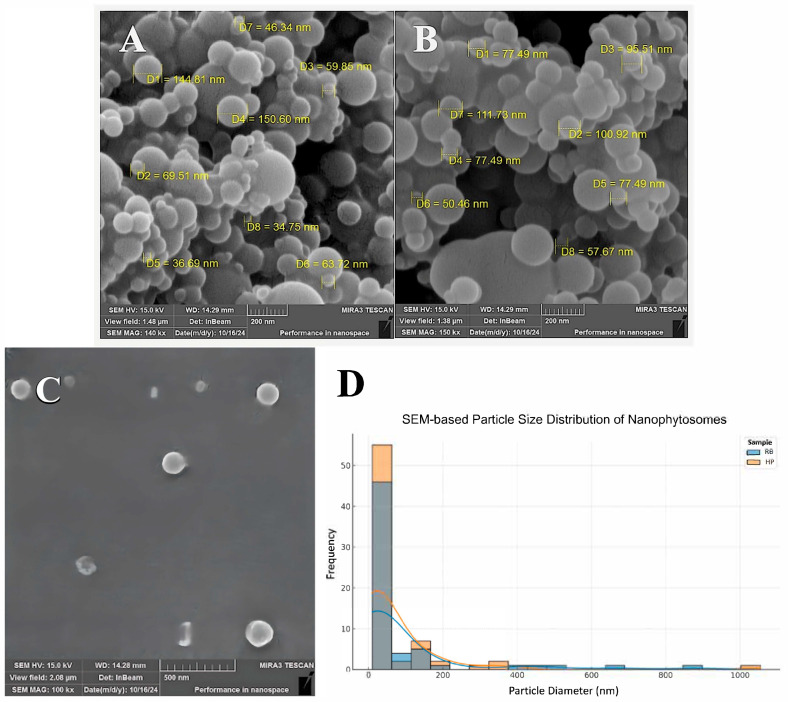
The SEM images of RB nanophytosomes (**A**); HP nanophytosomes; (**B**) and empty nanophytosomes (**C**). Scale bars are 200 nm for (**A**,**B**) and 500 nm for the image (**C**). Particle size distribution of nanophytosomes based on SEM images using ImageJ software version v2 1.4.7 (**D**).

Figure 2 illustrates the morphology and size analysis of RB nanophytosomes (A), HP nanophytosomes (B), and empty nanophytosomes (C), all of which display spherical particles indicating successful encapsulation of plant extracts. In order to achieve better insight into the particle size distribution in nanophytosome formulations, statistical evaluation through ImageJ software version v2 1.4.7 was performed according to SEM images (Figure 2D). The diameters of 61 individual RB nanophytosomes and 70 HP nanophytosomes were manually counted. The mean particle diameter was determined as 81.64 ± 161.99 nm for RB nanophytosomes and 66.57 ± 141.31 nm for HP nanophytosomes. Curiously, both of the samples had a wide particle size distribution, featuring particles above 800 nm (RB) and 1000 nm (HP). Such observations highlight the requirement of statistical sampling to capture nanoparticle populations suitably. The wide range of particle sizes might be due to aggregation, heterogenous synthesis, or a drying artifact from SEM imaging. These findings indicate the need to further optimize processing and formulation parameters to obtain more even particle size distributions.

The average sizes of the nanoparticles fall within an optimal range for drug delivery, demonstrating effective encapsulation and stability. According to DLS results, HP nanophytosomes measure 151 nm with a PDI of 0.147, and RB nanophytosomes are 148 nm with a PDI of 0.234. These findings align with the larger particles observed in electron microscopy images, confirming the polydisperse nature of RB nanophytosomes. Prior zeta potential tests indicated sufficient negative surface charge, promoting electrostatic repulsion and minimizing aggregation, which is further supported by SEM images showing a largely non-aggregated dispersion. The more consistent size and stability of HP nanophytosomes may be due to higher encapsulation efficiency. These results align with previous studies on herbal nanophytosomes, which typically achieve optimized particle size, improved dispersion, stability, and bioavailability for medicinal applications. Particle sizes between 50 and 200 nm are common in plant-based nanophytosome research, enhancing bioavailability and enabling targeted drug delivery. Particles under 150 nm are particularly favored for effective cellular uptake, corroborating findings that processing conditions and extract composition influence particle size during encapsulation [42].

### 3.3. Fourier Transform Infrared Spectroscopy (FTIR)

**Figure 3:**
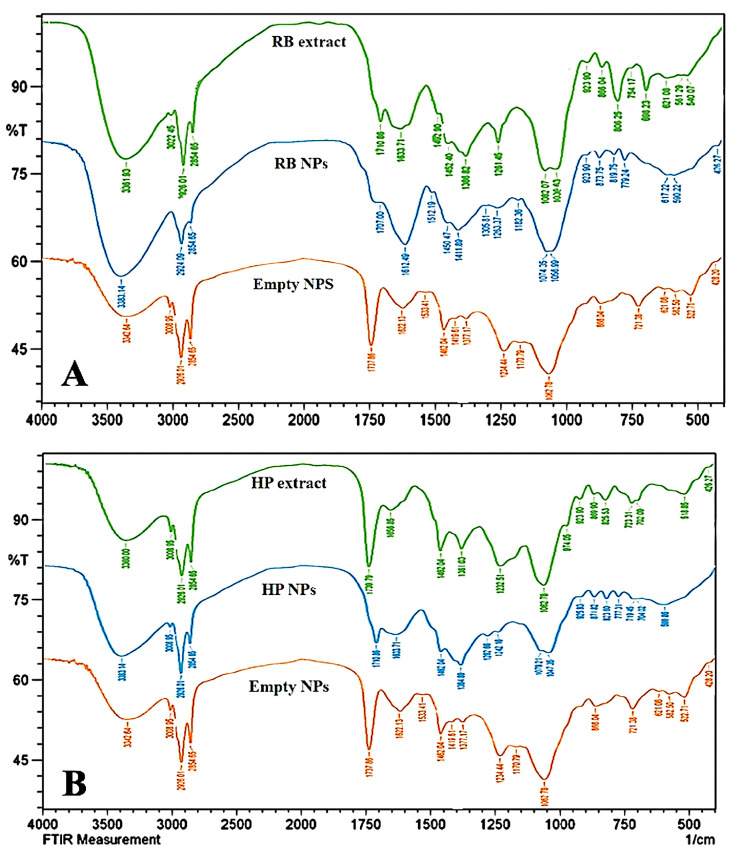
The FT-IR spectroscopic analysis of RB extract and nanophytosomes (**A**); the FT-IR spectroscopic of HP extract, HP nanophytosomes, and empty nanophytosomes (**B**). NPs: nanoparticles.

FTIR analysis of RB reveals various functional groups linked to key bioactive phytochemicals (Figure 3A). The broad peak at 3361.93 cm^−1^ indicates O-H stretching typical of hydroxyl groups in alcohols, phenols, or acids, which contribute to antioxidant properties in plants [43]. Peaks at 3022.45 cm^−1^ and 2926.01 cm^−1^ correspond to C-H stretching, with the former associated with aromatic C-H bonds and the latter with aliphatic C-H bonds [44]. The peak at 2854.65 cm^−1^ suggests the presence of -CH2 groups, indicating aliphatic chains in the extract [45]. A sharp peak at 1710.86 cm^−1^ likely represents C=O stretching from carbonyl groups in carboxylic acids, aldehydes, or esters, hinting at flavonoids or other pharmacologically active compounds [46]. C-O stretching peaks at 1039.03 cm^−1^ and 1082.97 cm^−1^ imply ethers, alcohols, or esters, while lower wavenumber peaks at 698.23 cm^−1^ and 754.17 cm^−1^ suggest aromatic ring out-of-plane bending [47,48]. Overall, the FTIR spectrum indicates the presence of alcohols, phenols, and, potentially, terpenoids, aligning with findings from other plant extracts rich in phenolic compounds. These phytochemicals may exhibit anti-inflammatory, antibacterial, and antioxidant activities. For example, studies on Euphorbia neriifolia and Cleome gynandra show similar C=O stretches, indicating bioactive compounds beneficial for pharmacological applications [49,50]. Thus, RB likely contains functional groups contributing to its medicinal value, reinforcing its potential use in folk medicine based on comparative phytochemical profiles. These spectral characteristics confirm the presence of polyphenolic compounds, particularly flavonoids and acids, which are responsible for the extract’s antioxidant activity. The observed bands provide a clear fingerprint of bioactive molecules in the RB extract.

The FTIR spectra of RB nanophytosomes reveal significant functional groups indicative of bioactive constituents from the plant (Figure 3A). The broad O-H stretch at 3383 cm^−1^ confirms phenolic or alcohol groups, while shifts in C-H stretching peaks (2924 cm^−1^, 2854 cm^−1^) and carbonyl stretching (1707 cm^−1^) suggest interactions between the nanophytosomes matrix and the RB extract [43,44]. These interactions may enhance the stability and bioavailability of the active compounds compared to the RB extract alone. Although peak intensities may vary, the main functional groups remain intact, indicating that while nanophytosomes modify the molecular environment, they preserve the extract’s bioactive components. Previous studies support that such encapsulation can improve the transport and therapeutic efficacy of these compounds, enhancing their anti-inflammatory and antioxidant properties [51]. Similar findings have been reported in encapsulated extracts of Ginkgo biloba and Curcuma longa, where FTIR analysis shows that bioactive compounds are effectively preserved within the nanophytosome system [52,53]. The minor shifts in O-H and C=O stretching peaks indicate successful interaction between the RB extract and the lipid bilayer. These changes suggest that encapsulation preserves the functional groups while enhancing molecular interactions and potential bioavailability.

The FTIR spectrum (Figure 3A) reflects the structural elements of the lipid bilayer of the empty nanophytosomes. The major components of the nanophytosomes are phospholipids, which indeed express many key peaks in the spectrum. The peak at 3342 cm^−1^ represents O-H stretching vibrations connected to hydroxyl groups. These can be assigned to water molecules or polar heads of phospholipids due to the outer bilayer of nanophytosomes [43]. The presence of O-H stretching verifies the typical hydrophilic contacts of the lipid-based carriers. Peaks at 2926 cm^−1^ and 2854 cm^−1^ represent the C-H stretching of the aliphatic hydrocarbon chains of the fatty acid components of phospholipids common in most of the lipid structures [45]. The peaks represent the hydrophobic tails of the phospholipid molecules. The band at 1737 cm^−1^ shows the C=O stretching of ester linkages in the fatty acid chains of phospholipids [46]. Bands at 1622 cm^−1^ and 1533 cm^−1^ are related to the amide I and II bands, respectively, which come from peptide bonds in proteins or similar structures [54]. If the phospholipid is made from lecithin or a related substance, it often contains amide groups. It should be noted that the peak at 1062 cm^−1^ shows stretching of the C-O-C or phosphate groups, which usually arises in the phospholipid backbones [47]. These lower peaks (866, 721, and 628 cm^−1^) might correspond to specific bending vibrations of either the backbone groups or aliphatic chains in the nanophytosomes’ structure [48].

The FTIR spectra indicate a well-ordered lipid bilayer structure, with significant peaks at phosphate, C-H, and C=O stretching, consistent with findings from Kim et al. (2019) [18] for empty nanophytosomes. Overlapping peaks in the 1500–500 cm^−1^ range and around 3400 cm^−1^ suggest common components like polymers or phospholipids in the nanophytosomes’ formulation [55]. The 3400 cm^−1^ peak, attributed to O-H stretching, indicates hydroxyl groups in the phospholipid head groups, while the peak near 1700 cm^−1^ likely reflects C=O stretching in amide or ester groups. Peaks at 1400 cm^−1^ and 1100 cm^−1^ may signify interactions between the nanophytosomes and bioactive compounds from the RB extract, suggesting successful encapsulation [56]. Similarities in FTIR spectra of empty and extract-loaded nanophytosomes have been reported in other studies, confirming the preservation of nanocarrier structures while indicating shifts that denote phytochemical incorporation [57]. The spectral features are consistent with phospholipid-based nanocarriers, showing characteristic lipid and amide bands. These peaks serve as a reference for identifying molecular interactions after plant extract loading.

The FTIR spectrum of HP extract (Figure 3B) shows key peaks indicating various functional groups. A peak at 3360 cm^−1^ likely corresponds to O-H stretching from hydroxyl groups in alcohols, phenols, or carboxylic acids, possibly due to water or polyphenolic compounds [43]. The C-H stretching vibrations at 2926 and 2854 cm^−1^ suggest the presence of lipids or fatty acids [44]. A strong peak at 1739 cm^−1^ indicates C=O stretching from carbonyl groups, associated with esters, aldehydes, or ketones in plant lipids or flavonoids [46]. The peak at 1656 cm^−1^ may result from N-H bending or C=C stretching, linked to carotenoids or proteins [58]. CH_2_ bending is indicated by the peak at 1462 cm^−1^, common in fatty acids [59]. Peaks at 1232 cm^−1^ and 1062 cm^−1^ suggest C-O stretching, indicating ethers, esters, or polysaccharides like cellulose or pectin [47]. The absorption at 974 cm^−1^ indicates out-of-plane C-H bending, possibly from unsaturated hydrocarbons or aromatic compounds [48]. Additionally, a peak at 518 cm^−1^ may reflect halogen-containing compounds or inorganic skeletal vibrations [60]. Overall, the HP extract contains functional groups, such as hydroxyl, carbonyl, and aliphatic chains, indicating the presence of fatty acids, polysaccharides, and phenolic compounds [61,62]. This profile aligns with known chemical characteristics of plant extracts and correlates with bioactive compounds found in Glaucum flavum [63]. The spectrum suggests the presence of phenolics, fatty acids, and polysaccharides, contributing to the extract’s therapeutic properties. These peaks form the chemical basis for the expected antioxidant and anti-inflammatory effects.

The FTIR spectrum of HP nanophytosomes, shown in Figure 3B, reveals key peaks similar to those in the HP extract. The peak at 3338 cm^−1^ indicates O-H stretching from hydroxyl groups, likely linked to lipid head groups or water in the nanophytosomes [43]. Peaks at 2926 cm^−1^ and 2854 cm^−1^ reflect C-H stretching from aliphatic chains, which is sharper in the nanophytosomes due to contributions from the lipid bilayer [44]. A broad band at 1710 cm^−1^ corresponds to C=O stretching, likely from ester bonds in phospholipids used in nanophytosomes preparation [46]. The peak at 1633 cm^−1^ suggests proteins or conjugated alkenes, indicating a shift from 1656 cm^−1^ in the HP extract due to encapsulation effects [58]. C-O stretching at 1078 cm^−1^ may arise from interactions between polysaccharides or esters and the lipid carrier [47]. Both spectra show a prominent peak around 3300 cm^−1^ for hydroxyl groups, which is sharper in the nanophytosomes due to interactions with the lipid bilayer [46]. The C=O stretching peak shifts from ~1739 cm^−1^ in the extract to 1710 cm^−1^ in the nanophytosomes, suggesting interaction with the phospholipid bilayer [46]. The polysaccharide and ester region also shows a shift (1062 cm^−1^ vs. 1078 cm^−1^), indicating more ordered interactions post-encapsulation [47]. Overall, sharper and better-defined peaks in the HP nanophytosomes suggest enhanced molecular interactions, consistent with findings from other studies on lipid-based carriers containing plant extracts [64]. Slight shifts in carbonyl and O-H bands indicate non-covalent interactions with the phospholipid matrix. The changes confirm the encapsulation and improved structural organization of the HP extract within the nanocarrier system [65].

The FTIR spectra reveal notable similarities between the significant peaks of empty nanophytosomes and HP nanophytosomes (Figure 3B). The O-H stretching appears at 3342 cm^−1^ for empty nanophytosomes and 3338 cm^−1^ for HP nanophytosomes, suggesting a change in the hydrogen-bonding environment due to interactions with the lipid bilayer [43]. C=O stretching is observed at 1737 cm^−1^ in empty nanophytosomes, with a shift in the HP nanophytosomes indicating interaction between phospholipid carbonyl groups and the encapsulated extract [46]. Amide I and II bands shift from 1622 cm^−1^ and 1533 cm^−1^ in empty nanophytosomes to 1633 cm^−1^ and 1462 cm^−1^ in HP nanophytosomes, highlighting enhanced interactions from the plant extract [54]. The strong C-H stretching bands at 2926 cm^−1^ and 2854 cm^−1^ are more intense in HP nanophytosomes, reflecting additional hydrocarbon interactions with phytoconstituents [44]. C-O stretching around 1062 cm^−1^ indicates the phospholipid backbone [47]. Overall, significant shifts in functional group peaks suggest successful encapsulation through non-covalent interactions, confirming increased stability consistent with recent studies on lipid-based nanocarriers. Comparative shifts in key functional groups (e.g., C=O and amide bands) further support the successful incorporation of HP extract into the lipid structure. The findings align with previous FTIR studies on phytosome-based delivery systems [65].

### 3.4. Drug Loading (DL) and Encapsulation Efficiency (EE) Studies

Both RB and HP extracts demonstrated effective encapsulation in nanophytosomes, with drug loading (DL) values of 41 ± 0.35% for RB and 48 ± 0.78% for HP. The DL values were calculated using the total formulation weight, including both phospholipid and encapsulated extract. The higher DL for HP indicates greater compatibility with the nanophytosomes matrix, likely due to differences in solubility and chemical composition of the extracts. The encapsulation efficiency (EE) was also high, at 77 ± 1.78% for RB and 81 ± 2.05% for HP, suggesting that the nanophytosomes can effectively retain these extracts, enhancing their bioavailability and therapeutic effects. Both nanophytosome types may sustain therapeutic concentrations over extended periods, benefiting drug delivery with lower dosing frequency. The EE values achieved are higher than those reported in previous studies, indicating the effectiveness of the developed methodology for various medicinal plant extracts [66]. The differing chemical properties of the extracts further explain the superior encapsulation performance of HP compared to RB, aligning with findings from similar research on extract composition and encapsulation capabilities [67].

### 3.5. Release Profile of Nanophytosomes

**Figure 4:**
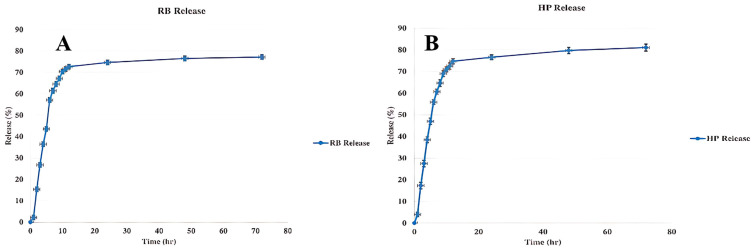
(**A**) Release of RB from the nanophytosomes. (**B**) Release of HP from the nanophytosomes. Results are shown as mean ± SD (*n* = 3). Statistical differences were assessed through one-way ANOVA with a comparisons test (*p* < 0.05).

The release characteristics of plant extracts encapsulated in RB nanophytosomes (Figure 4A) and HP nanophytosomes (Figure 4B) were evaluated using the dialysis bag method. The drug release profile of nanophytosomes is shown as the mean ± SD (*n* = 3). Statistical differences were assessed through one-way ANOVA with a comparisons test (*p* < 0.05). Both graphs show an initial burst release of about 70–80% within the first few hours, followed by a steady, prolonged release phase. This biphasic release pattern is typical for nanophytosomes produced via solvent evaporation, where the initial burst is due to surface diffusion, while the sustained release results from slower diffusion from within the nanophytosomes. The observed burst release (70–80%) is typical for nanocarriers containing surface-associated drugs. Future formulation optimization, such as increasing the lipid-to-drug ratio, may help reduce the initial burst and enhance sustained release for applications requiring controlled delivery. Similar studies on nanocarriers, such as those loaded with Camellia sinensis extract, have demonstrated that this release pattern facilitates rapid therapeutic response and extended activity [68]. Given the comparable release profiles of RB and HP extracts, it is likely that both formulations exhibit similar encapsulation stability and release characteristics due to their analogous lipid content and encapsulation efficiencies. Both nanophytosomes achieve around 80–85% release during the test period, aligning with findings from other plant-extract-loaded nanocarriers that emphasize enhanced bioactivity through effective encapsulation and prolonged release [69]. The extended-release capability significantly boosts the therapeutic potential and bioavailability of medicinal plant extracts compared to conventional formulations.

### 3.6. Cytotoxicity Evaluations

**Figure 5:**
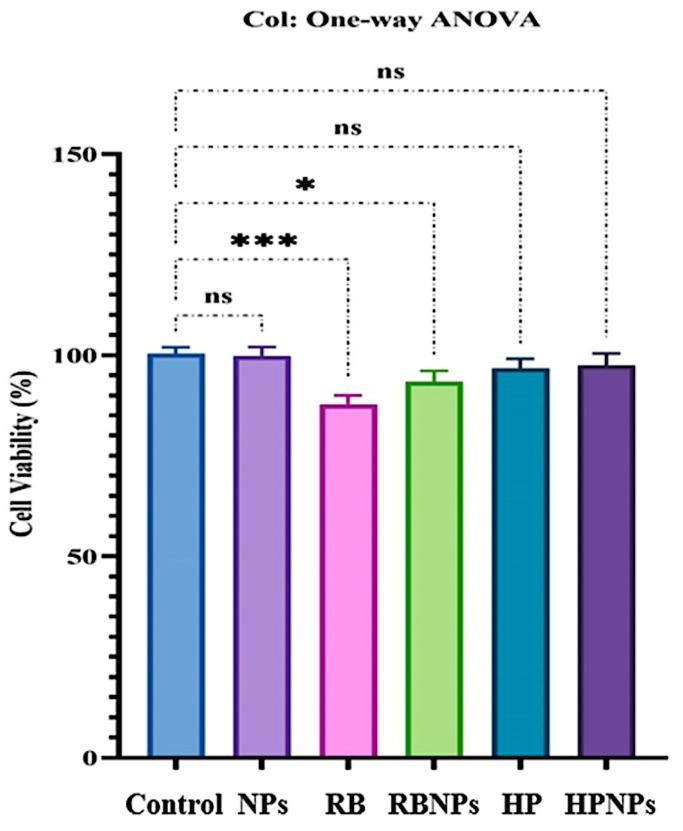
Cell viability percentage of fibroblast cell line after treatment with the highest concentrations of RB and HP samples loaded in nanophytosomes. The error bars in all columns are reported as the mean ± SEM (*p* value < 0.05). * *p* < 0.01, *** *p* < 0.0001, ns: not significant.

The cytocompatibility of sample nanophytosomes (NPs), nanophytosomes with RB (RB NPs), and HP extracts (HP NPs) and the extracts alone (RB and HP) in nanophytosomes was assessed using the MTT assay on HSF-PI 16 cells at concentrations ranging from 10 to 200 µg/mL for 24 h (Figure 5). Our results indicated that the high concentration (200 µg/mL) of the RB and RB nanophytosome sample groups led to a significant decrease in the viability percentage of fibroblast cell lines compared to the control group (87.74% and 93.42% vs. 100%). At 200 µg/mL, RB extract led to a decrease of about 12% in fibroblast viability. However, cell viability remained above 85%, which is considered acceptable according to regulatory biocompatibility guidelines. Additionally, the high concentrations (200 µg/mL) of nanophytosome, HP, and HP nanophytosome sample groups also showed a reduction in fibroblast cell line viability percentages compared to the control group (99.82%, 96.67%, and 97.52% vs. 100%), although these reductions were not statistically significant. Consequently, the HP samples loaded in nanophytosomes at concentrations up to 200 µg/mL demonstrated cytocompatibility, supporting their safety for potential in vivo biomedical applications.

### 3.7. Stability Studies

**Figure 6:**
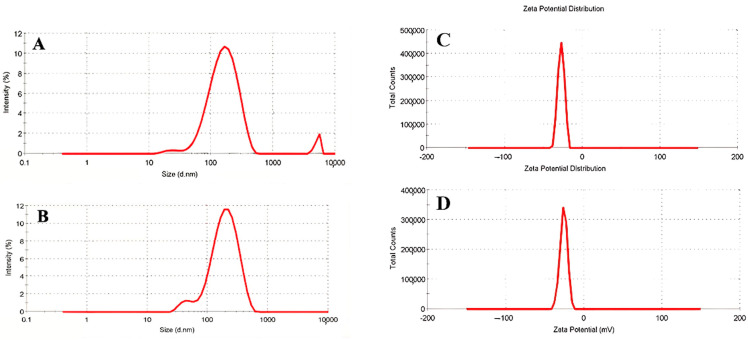
DLS analysis of RB nanophytosomes after 30 days (**A**). DLS analysis of HP nanophytosomes after 30 days (**B**), the zeta potential of RB nanophytosomes after 30 days (**C**), and the zeta potential of HP nanophytosomes after 30 days (**D**). Both samples were stored at room temperature (25 ± 2 °C).

Nanocarriers, such as RB nanophytosomes and HP nanophytosomes, often experience slight increases in size over time due to aggregation or structural changes. DLS data showed that after 30 days, the size of RB nanophytosomes increased from 148 to 152.8 nm (Figure 6A), while HP nanophytosomes grew from 151 to 160.5 nm (Figure 6B). The 4.8 nm increase in RB nanophytosomes indicates good stability, as it falls within an acceptable range. Minor size increases can occur due to processes like particle fusion or water absorption. In comparison to other nanophytosome systems that have shown size increases of 10 to 20 nm due to instability, the slight rise in RB nanophytosomes suggests that they maintain stability over time [70].

The HP nanophytosomes exhibited a slightly larger increase of 9.5 nm, which may indicate greater particle interactions or different interactions with the lipid membrane due to the hydrophobic nature of the HP extract. Possible causes for size increases in both formulations include aggregation and osmotic swelling, which can lead to structural modifications of the nanophytosomes [71,72]. Similar studies with plant-extract-loaded nanophytosomes have reported size increases of 5–10 nm after 30 days, with resveratrol-treated nanophytosomes showing up to 15 nm increases [73]. Overall, these findings suggest that size changes between 5 and 10 nm are typical and indicative of stable nanophytosome formulations [74,75]. Furthermore, post-storage zeta potential values remained within a similar range of −24 mV for RB nanophytosomes and −26 mV for HP nanophytosomes (Figure 6C,D), confirming maintained colloidal stability over 30 days.

## 4. Conclusions

In this study, we successfully developed nanophytosomes containing extracts from two medicinal plants, HP and RB, marking the first instance of their use as safe nanocarriers to enhance the bioavailability and stability of secondary metabolites. Additionally, the physicochemical properties of these lesser-known plant extracts were thoroughly examined. The findings revealed that the extracts are abundant in various phenolic compounds and possess antioxidant, anti-inflammatory, and antibacterial properties, making them promising candidates for medicinal applications. The synthesized nanophytosome demonstrates potential for improved stability and bioavailability of plant metabolites, warranting further in vivo evaluation. Furthermore, cytotoxicity assessments confirmed that the nanocarrier is safe and non-toxic. Consequently, this phytosomal formulation can be utilized as a safe candidate nanocarrier to enhance bioavailability, improve stability, and mitigate the side effects of plant metabolites. These nanocarriers represent a novel strategy for therapeutic applications and herbal bioactive-based drug delivery, thereby enhancing the effectiveness of traditional medicine.

## Data Availability

The original contributions presented in this study are included in the article. Further inquiries can be directed to the corresponding author.
